# Inappropriate screening of obstructive coronary artery disease during pre-anesthesia assessment of candidates for non-cardiac surgery

**DOI:** 10.1590/1414-431X202010466

**Published:** 2021-01-08

**Authors:** A.C.C. Oliveira, L.A. dos Santos, L.B. da Silva, J.R.P. Lopes, P.A. Schwingel, L.C.L. Correia

**Affiliations:** 1 Programa de Pós-Graduação em Medicina e Saúde Humana, Escola Bahiana de Medicina e Saúde Pública, Salvador, BA, Brasil; 2 Serviço de Anestesiologia, Complexo Hospitalar Universitário Professor Edgard Santos, Universidade Federal da Bahia, Salvador, BA, Brasil; 3 Laboratório de Pesquisas em Desempenho Humano, Universidade de Pernambuco, Petrolina, PE, Brasil

**Keywords:** Preoperative period, Perioperative care, Routine diagnostic tests, Operative surgical procedures

## Abstract

Preoperative evaluation in elective surgeries has been associated with successful surgical treatment. However, there is no solid scientific evidence that screening for coronary artery disease (CAD) reduces surgical risk. The aims of this study were to describe the frequency of inappropriate investigation of obstructive CAD induced by pre-anesthetic assessment in individuals without cardiovascular symptoms (candidates for low- to intermediate-risk surgeries) and to evaluate predictors of this conduct. We performed a retrospective evaluation of medical records of anesthesiology services from patients undergoing pre*-*anesthesia assessment between May 2015 and May 2016, including those with functional capacity ≥4 metabolic equivalents without a diagnosis of heart disease. A total of 778 medical records (47±16 years of age, 62.6% female) were studied. A private hospital performed 50.1% of the surgeries and 60.4% were of intermediate risk. Only 2.7% (95%CI: 1.7-4.1%) were screened for CAD, and 91% of these requests were mediated by cardiology consultations performed during pre-anesthetic testing visits. Factors associated with screening for CAD were hypertension, diabetes, moderate systemic disease (ASA III), cardiac consultation, previous diagnosis of CAD, and admission to a private hospital. Independent predictors were private hospitals (OR: 3.9; 95%CI: 1.3-11.0), ASA III (OR: 5.3; 95%CI: 1.7-16.2), and hypertension (OR: 3.8; 95%CI: 1.5-9.8). The frequency of inappropriate requests for CAD screening in asymptomatic individuals without untreated systemic diseases was low in pre-anesthetic visits. Although infrequent, screening for CAD is more common in the private setting, in patients with poorer health status, and is usually prescribed during cardiology consultation.

## Introduction

Data recorded in the information system of the Brazilian Unified Health System (SUS) show that throughout 2019, approximately four million patients underwent surgical procedures in Brazil, with elective surgeries accounting for more than 2.5 million of them (1). Therefore, given the high prevalence of heart disease in the general population, almost a third of these patients have either coronary artery disease (CAD) or CAD risk factors (2). The prevalence of clinical CAD is around 10% (3) in developed or developing countries, and the incidence and severity of CAD increases with age in both sexes, increasing the frequency of surgeries (4–6). Thus, the incidence of adverse cardiac events, such as acute myocardial infarction and death from cardiac causes after non-cardiac surgery may reach 3.9%, particularly in major surgeries (7,8).

Pre-anesthetic evaluation aims to minimize the risk of perioperative surgical complications, by planning intraoperative trans-care to prevent cardiovascular events associated with hypotension, volume overload, and acute anemia (8). A common practice emerging from these evaluations is the pursuit of the diagnosis of silent coronary disease (screening), motivated by the belief that this would generate cardiovascular protection for patients. Although this approach generates a sense of safety in patients with negative results, positive results promote a cascade of conduct that not only fail to attenuate the risk of cardiovascular events (7,9,10), but may lead to undesirable outcomes, ranging from suspension of surgery to inappropriate revascularization and adverse events resulting from overdiagnosis and overtreatment (11). Therefore, surgeons and anesthesiologists often consult with cardiologists during this assessment to reduce serious cardiac events (12).

Thus, CAD screening is deemed inappropriate in minor and medium complexity elective surgeries when patients are asymptomatic and have at least moderate functional capacity and eventual systemic diseases under clinical control (13,14). The screening does not effectively contribute to preoperative evaluation nor reduces the risk of cardiac complications in the perioperative period and may be potentially harmful to the patients' physical and mental health (14–16).

Therefore, the present study aimed to describe the frequency of inappropriate investigation of obstructive CAD induced by pre-anesthetic assessment in individuals without any cardiovascular symptoms, candidates for minor and medium complexity non-cardiac surgeries, and to evaluate potential outcomes of this approach.

## Material and Methods

### Study design

This was an observational, cross-sectional study, with data collected from medical records of the Hospital Universitário Professor Edgard Santos (public) and Hospital Jorge Valente (private, with no teaching activity), both in Salvador, Bahia, Brazil.

This research was approved by the Research Ethics Committee (CEP) of the Escola Bahiana de Medicina e Saúde Pública (EBMSP) under CAAE 57161016.8.0000.5544 and by the CEP of the Hospital Universitário Professor Edgard Santos (HUPES) under CAAE 57161016.8.3001.0049. Due to the retrospective nature of the study, both CEPs waived the informed consent form.

### Sample selection

Medical records of patients who underwent pre-anesthetic consultation between May 2015 and May 2016 were analyzed. The following inclusion criteria were considered: candidates for low- to intermediate-risk surgeries, asymptomatic for cardiovascular diseases, functional capacity ≥4 metabolic equivalents (MET), and with no history of untreated systemic diseases.

Endoscopic procedures, superficial procedures, cataract, breast, or outpatient surgeries were classified as minor elective surgeries (risk of perioperative mortality <1%) (17). Intraperitoneal, intrathoracic, carotid endarterectomy, head and neck, orthopedic, and prostate surgeries were classified as medium complexity surgeries (risk of perioperative mortality between 1 and 5%).

In the two anesthesiology services analyzed, medical records provided information on functional capacity in a parameterized manner according to the Duke Activity Status Index (DASI), a questionnaire with 12 items assessing daily activities, such as personal hygiene, locomotion, domestic tasks, sexual function, and recreation with the respective metabolic costs (18). Each item carries a specific weight based on the metabolic cost. Participants are asked to identify which of the activities listed they can perform. The final DASI score varies between zero and 58 points, and the higher the score, the better the functional capacity. It is worth emphasizing that positive responses to the first four DASI items estimate functional capacity at more than 4 MET.

We excluded medical records lacking relevant information on pre-anesthetic evaluations, return visits with other doctors (inter-consultations), and reports of complementary exams.

### Data collection

Data collection was performed by reviewing medical records. The main variable analyzed was obstructive CAD screening as part of preoperative assessment, defined by the following tests: exercise stress test, stress myocardial perfusion scintigraphy, echocardiogram under pharmacological or physical stress, coronary angiotomography, or coronary angiography.

Clinical and anthropometric data and physical status according to the American Society of Anesthesiology (ASA) (19) were collected and analyzed, along with high-risk comorbidities for obstructive CAD, surgical topography, and surgical extent, and the type of hospital (public or private).

### Data analysis

The data obtained were double entered into the SPSS computer program (IBM, version 16.0.2, 2008, USA), with verification of consistency and range. Descriptive statistical analysis was performed with categorical variables presented as absolute and relative frequencies, while continuous variables are reported as means±SD after data normality was determined by the Kolmogorov-Smirnov test. Alanine aminotransferase and creatinine values are reported as median (first quartile [1Q]-third quartile [3Q]).

The frequency of CAD screening was described in percentage and inaccuracy was quantified by a 95% confidence interval (CI). In the univariate analysis, individuals with and without CAD screening were compared regarding numerical variables using Student's *t*-test, and for categorical variables using Pearson's chi-squared test (χ^2^). Variables with P≤0.20 in the univariate analysis were selected for multivariate analysis by logistic regression, performed by the stepwise technique, to identify predictors of inappropriate CAD screening. Unadjusted and adjusted odds ratios (OR) and 95%CI were calculated. Statistical analyzes were two-tailed, and statistical significance was defined by P<0.05.

## Results

### Sample features

A total of 800 medical records were screened, of which 10 (1.2%) were excluded due to lack of a pre-anesthetic evaluation form, eight (1.0%) due to the absence of an inter-consultation report, while four (0.5%) records did not have the complementary examination reports requested. Thus, 778 medical records of asymptomatic patients who were candidates for non-cardiac surgeries and had undergone pre-anesthetic evaluation were analyzed. Patients’ age was 47±16 years, 63% women, 60% underwent medium complexity surgeries, and 50% were recruited from a private hospital. Other characteristics are described in Table 1.

**Table 1:** Characterization of patients included in the study from Salvador, BA, Brazil (n=778).

Variables	n (%)
Hospital	
Public	388 (50%)
Private	390 (50%)
Physical status	
Healthy (ASA I)	294 (38%)
Mild or controlled systemic disease (ASA II)	417 (54%)
Moderate systemic disease (ASA III)	67 (8.6%)
Complexity of the surgery	
Minor	308 (40%)
Medium	470 (60%)
Surgical topography	
Head and neck surgery	98 (13%)
Thoracic surgery	9 (1.2%)
Abdominal surgery	176 (23%)
Pelvic surgery	127 (16%)
Orthopedic surgery	82 (11%)
Endoscopic procedure	76 (9.8%)
Cardiology consultation	142 (18%)
Abnormal chest x-ray	39 (6.8%)
Abnormal electrocardiogram (ECG)	53 (8.3%)
Arterial hypertension	243 (31%)
Diabetes mellitus	85 (11%)
Coronary artery disease	20 (2.6%)

ASA: American Society of Anesthesiology.

Mean body mass index (BMI) was 27±5 kg/m^2^, mean systolic and diastolic blood pressure was 126±18 mmHg and 80±11 mmHg, respectively, and median value (1Q-3Q) of creatinine was 0.8 (0.7-0.9) mg/dL (Table 2).

**Table 2:** Clinical and anthropometric data of patients scheduled for low and medium risk surgeries with moderate functional capacity (≥4 MET, metabolic equivalents), in Salvador, BA, Brazil (n=778).

Variables	Mean±SD
Age (years)	47.3±15.7
Total body mass (kg)	73.0±14.9
Body mass index (kg/m^2^)	26.6±5.0
Systolic blood pressure (mmHg)	126.2±18.0
Diastolic blood pressure (mmHg)	79.9±11.5
Axillary temperature (°C)	36.3±1.5
Resting heart rate (bpm)	76.0±10.4
Respiratory rate (rpm)	18.4±1.5
Hemoglobin (g/dL)	13.0±1.8
Hematocrit (%)	39.4±5.0
Platelet count (n)	250,682±77,371
Fasting glucose (mg/dL)	94.6±19.0
Prothrombin activity (%)	91.4±12.6
International normalized ratio (INR)	1.06±0.35
Activated partial thromboplastin time (s)	32.7±4.7
Aspartate aminotransferase (mg/dL)	27.7±8.6
Alanine aminotransferase (mg/dL)*	37.0 (29.0-45.0)
Urea (mg/dL)	31.7±14.8
Creatinine (mg/dL)*	0.8 (0.7-0.9)

*Median (first quartile-third quartile).

### Inappropriate CAD screening

Only 21 patients underwent CAD screening during pre-anesthetic evaluation, corresponding to a prevalence of 2.7% (95%CI: 1.7-4.1%) of inappropriate screening for obstructive CAD in this population (Table 3). Most of these procedures were mediated by cardiology consultation, with the examination requested by the anesthesiologist in only 10% of cases (2 patients). The univariate analysis showed a potential association between CAD screening and the following variables: private hospital (P<0.048), consultation with a cardiologist (P=0.001), patients with ASA III physical status (P=0.001), and patients with the presence of stable CAD (P=0.04). In addition, patients surveyed for CAD had higher mean age (P<0.001), total body mass (P=0.003), and BMI (P=0.04).

**Table 3:** Association between the studied parameters and screening for coronary artery disease (CAD) in asymptomatic patients scheduled for minor and medium-risk surgeries with moderate functional capacity (≥4 MET, metabolic equivalents) in Salvador, BA, Brazil (n=778).

Variables	CAD screening		P
	Yes (n=21)	No (n=757)	
Private hospital, n (%)	15 (71%)	375 (47%)	0.048
Male gender, n (%)	12 (57%)	279 (37%)	0.058
Age (years)	59.3±16.0	46.9±15.5	<0.001
Total body mass (kg)	81.6±17.2	72.8±14.9	0.003
Body mass index (kg/m^2^)	29.3±5.6	26.8±5.0	0.036
Systolic blood pressure (mmHg)	131.9±14.6	126.1±18.2	0.150
Diastolic blood pressure (mmHg)	79.7±10.3	79.9±11.6	0.919
Moderate systemic disease (ASA III), n (%)	6 (29%)	61 (8.1%)	<0.001
Cardiology consultation, n (%)	19 (90%)	115 (15%)	<0.001
Systemic arterial hypertension, n (%)	14 (67%)	229 (30%)	<0.001
Diabetes mellitus, n (%)	7 (33%)	78 (10%)	<0.001
Coronary artery disease, n (%)	2 (9.5%)	18 (2.4%)	0.041

Data are reported as absolute and relative frequencies or means±SD. Statistical analysis was carried out using Pearson's chi-squared test, Fisher's exact test, or Student's *t*-test. ASA: American Society of Anesthesiology.

These variables were entered into a multivariate analysis that showed the following independent predictors of inappropriate CAD screening: private hospitals (OR: 3.9; 95%CI: 1.3-11.0), patients with ASA III physical status (OR: 5.3; 95%CI: 1.7-16.2), and systemic arterial hypertension (OR: 3.8; 95%CI: 1.5-9.8) (Table 4). Screening of CAD in private hospitals totaled 3.8% (95%CI: 2.3-6.3) and in the university hospital, 1.5% (95%CI: 0.6-3.4). In ASA III patients, frequency was 9.0% (95%CI: 3.8-18.5), which was higher than the 2.1% (95%CI: 1.3-3.5) found in the other categories. Hypertensive individuals screened for CAD accounted for 5.8% patients (95%CI: 3.4-9.5), while non-hypertensive patients were only 1.3% (95%CI: 0.6-2.7).

**Table 4:** Multivariate analysis of factors associated with coronary artery disease (CAD) screening in asymptomatic patients scheduled for low and medium-risk surgeries with moderate functional capacity (≥04 MET, metabolic equivalents) in Salvador, BA, Brazil (n=778).

Variables	Not adjusted		Adjusted	
	OR (95%CI)	P	OR (95%CI)	P
Private hospital	3.16 (1.03−9.67)	0.044	3.90 (1.36−11.00)	0.011
Female gender	2.34 (0.88−6.24)	0.090	−	−
ASA III physical status	3.13 (0.86−11.4)	0.083	5.30 (1.70−16.20)	0.003
Hypertension	2.77 (0.93−8.25)	0.068	3.79 (1.50−9.80)	0.006
Diabetes mellitus	1.84 (0.59−5.78)	0.293	−	−
Coronary artery disease	1.83 (0.31−10.8)	0.508	−	−
Age	1.02 (0.98−1.05)	0.286	−	−
Body mass index	1.06 (0.96−1.17)	0.231	−	−

ASA (American Society of Anesthesiology) III: moderate systemic disease; OR: odds ratio; CI: confidence interval.

## Discussion

Randomized clinical trials have consistently demonstrated the lack of clinical effectiveness of coronary disease screening in the preoperative period of a non-cardiac surgery (20). This was a cross-sectional study to describe the prevalence of CAD screening in asymptomatic candidates for minor and medium complexity surgeries, which is considered inappropriate based on previous scientific evidence (13–16). The present study found a low prevalence of inappropriate CAD screening in asymptomatic patients who underwent pre-anesthetic consultation undergoing low-risk or intermediate non-cardiac surgeries. Screening for CAD was by far prescribed by cardiologists; only a minority of the patients was screened by the prescription of anesthesiologists. This suggests that the phenomenon of coronary disease over-diagnosis, apparently common in the preoperative period of surgery, is not mediated by pre-anesthetic evaluation.

According to the theory of behavioral economics (21,22), human behavior is based on mental shortcuts known as heuristics that lead to systematic cognitive biases that are far from a predictable and effective rational result. This reality also permeates the medical environment, and the prevailing paradigm ‘the more the better' remains on both sides of the equation, suppliers and consumers, even if the underlying motivations are from diametrically opposed spectra (22,23).

However, we did not find signs of medical overuse in the studied scenarios. Our findings were consistent with the body of evidence from the American public system in which Sheffield et al. (24) demonstrated a 3.8% prevalence of inappropriate performance of cardiac stress tests in 74,785 elective non-cardiac and non-vascular surgical patients, after surveying the Medicare database (1996 to 2008). Similarly, Kerr et al. (25) found 2.1% of cardiac tests by analyzing the Medicare database for patients undergoing minor surgeries in 2009. A review of patients who underwent cataract surgery and shoulder and knee arthroscopies in the same timeframe in the Veterans Affairs Corporate Data Warehouse have found that the prevalence of inappropriate cardiac stress tests in 22,697 veterans was also low (0.7%) (25).

In a sample of 5% of Medicare hospitalization requests between 1996 and 2008, with patients aged ≥66 years who underwent elective procedures of general surgery, urology, or orthopedics, 2,803 (3.8%) underwent preoperative cardiac stress tests without any indication (24). Our study corroborates these findings and extends this low frequency of investigation to the private environment. Although the private system has been a predictor of CAD screening with twice the requests of the public system, the absolute frequency of investigation remains low (15).

Additionally, the multivariate analysis revealed two other independent predictors of inappropriate CAD screening: patients with ASA III physical status and systemic arterial hypertension. Considering the multitude of variables related to the phenomenon, it is very strange that just three independent factors were verified. This fact probably reveals a lack of statistical power, considering we had only 21 outcomes, but it may reflect the reality, because anesthetic evaluation is strictly based on global surgical risk.

Requests for cardiovascular screening in patients without signs and symptoms of cardiovascular disease, routine assessment, and monitoring of heart disease without changes in clinical status, particularly in asymptomatic patients are considered inappropriate (26,27). Assessing the frequency of findings showing changes compatible with structural or functional heart disease in 1,071 echocardiogram exams performed at a private cardiology clinic in the countryside of the state of Bahia, Lopes et al. (28) identified that 53% of the requested examinations were inappropriate, since they would be less likely to generate beneficial effects than negative outcomes in the evaluated patients. In addition, they found that 9.4% of these tests were performed on asymptomatic patients during preoperative evaluation of a non-cardiac surgery.

A retrospective analysis of medical records of 154 patients undergoing obesity surgery at Cleveland Clinic Florida was carried out by Afolabi et al. (9), who identified that 25 (32%) of the 78 patients undergoing preoperative cardiac stress tests had positive results. All patients with positive stress test results underwent cardiac catheterization preoperatively, and cardiac angiography did not reveal significant obstructive CAD in 24 patients with abnormal findings in the study of nuclear stress. Only one patient had an obstructive lesion of the coronary artery on cardiac catheterization, requiring the placement of a coronary stent before the obesity surgery. In addition, there were five (3.2%) non-fatal cardiac events during surgery and the hospital mortality rate was 0%.

The fact that preoperative invasive procedures such as coronary artery bypass grafting or percutaneous coronary angioplasty do not reduce perioperative risk in patients with significant stable CAD is highlighted (7,10). These findings also reinforce that the request for these preoperative exams results in over-diagnosis with subsequent unnecessary treatments, without a solid evidence base that the outcome is positive considering benefits, costs, and possible damages (11,14,29).

Diagnostic overuse is commonly expected in risk mitigation scenarios and this justifies a universal tendency to screen for coronary disease. Given this finding, we must ask why anesthesiologists are immune to the inappropriate use of this diagnostic screening. First, in the pre-anesthetic consultation scenario, the primary goal of the assessment is to serve as a bridge to the surgical procedure. As the objective is surgery, an excess of diagnostic investigation would represent a barrier to the main medical action. This would also explain the low prevalence of referral for cardiology consultation. The physician's mental model in the pre-anesthetic consultation is surgical referral.

Although doctors could be affected by “attribution bias”, in this case the action is surgery and diagnostic overuse would promote surgical underuse. Therefore, viewing the surgery as an “end” would inhibit “half” of the procedures that are questionable. This is different from a routine clinical consultation when evaluation is the “end”. Thus, this study presents the following hypothesis: the prospect of a remote future procedure inhibits a futile procedure in the immediate future.

This hypothesis goes against the “bias of the present”, demonstrated by experiments in which individuals abdicate full pleasure in the future to anticipate partial pleasure. Children, for example, more commonly choose half a candy now than a whole candy in the future (30). Therefore, our second inference is that the present bias does not prevail in professional contexts, which is not exactly about pleasure itself, but the satisfaction emerging from effectively performing a professional intervention.

It is worth mentioning that the purpose of this cross-sectional study was only describing the prevalence of CAD screening; it was a study about the physician's preference, not about the patient's prognosis. We also did not perform a quantitative cost-benefit analysis for technical reasons; however, it could be evaluated in further studies. Finally, we must point out that the retrospective character of the study may be a limitation of our research, although the standardized approach of the anesthesiology teams facilitated data collection and analysis.

The present study suggested that the inappropriate screening for coronary artery disease was low in the pre-anesthetic evaluation context. Although infrequent, this practice predominantly occurs in private hospitals, with hypertensive patients, and with patients with overall poorer heath status.
